# Development of Toughened Flax Fiber Reinforced Composites. Modification of Poly(lactic acid)/Poly(butylene adipate-co-terephthalate) Blends by Reactive Extrusion Process

**DOI:** 10.3390/ma14061523

**Published:** 2021-03-20

**Authors:** Jacek Andrzejewski, Michał Nowakowski

**Affiliations:** 1 Polymer Processing Division, Institute of Materials Technology, Faculty of Mechanical Engineering, Poznan University of Technology, ul. Piotrowo 3, 61-138 Poznan, Poland; 2 MATRIX Students Club, Polymer Processing Division, Poznan University of Technology, ul. Piotrowo 3, 61-138 Poznan, Poland; michal.m.nowakowski@student.put.poznan.pl; 3 Faculty of Materials Engineering and Technical Physics, Poznan University of Technology, ul. Piotrowo 3, 60-965 Poznan, Poland

**Keywords:** poly(lactic acid), poly(butylene adipate-co-terephthalate), polymer blends, flax fiber composite, reactive extrusion, toughening, compatibilization

## Abstract

The presented study focuses on the development of flax fiber (FF) reinforced composites prepared with the use of poly(lactic acid)/poly(butylene adipate-co-terephthalate)—PLA/PBAT blend system. This type of modification was aimed to increase impact properties of PLA-based composites, which are usually characterized by high brittleness. The PLA/PBAT blends preparation was carried out using melt blending technique, while part of the samples was prepared by reactive extrusion process with the addition of chain extender (CE) in the form of epoxy-functionalized oligomer. The properties of unreinforced blends was evaluated using injection molded samples. The composite samples were prepared by compression molding technique, while flax fibers reinforcement was in the form of plain fabric. The properties of the laminated sheets were investigated during mechanical test measurements (tensile, flexural, impact). Differential scanning calorimetry (DSC) analysis was used to determine the thermal properties, while dynamic mechanical thermal analysis (DMTA) and heat deflection temperature (HDT) measurements were conducted in order to measure the thermomechanical properties. Research procedure was supplemented with structure evaluation using scanning electron microscopy (SEM) analysis. The comparative study reveals that the properties of PLA/PBAT-based composites were more favorable, especially in the context of impact resistance improvement. However, for CE modified samples also the modulus and strength was improved. Structural observations after the impact tests confirmed the presence of the plastic deformation of PLA/PBAT matrix, which confirmed the favorable properties of the developed materials. The use of PBAT phase as the impact modifier strongly reduced the PLA brittleness, while the reactive extrusion process improves the fiber-matrix interactions leading to higher stiffness and strength.

## 1. Introduction

In the case of many varieties of biopolymers, their performance does not match their petrochemical varieties. This applies to most of the mechanical, thermomechanical and processing properties. At present, the vast majority of biopolymers are used in the packaging industry for the production of films. Seemingly, the applications involving the production of films require only adequate puncture resistance and a high degree of elongation before tearing. However, in fact, materials in this category have to meet a number of special requirements such as optical properties, gas barrier and thermal properties. Rheological properties are also of particular importance, including characteristics, such as viscosity or melt strength [1,2,3,4]. Meeting such a wide range of requirements is usually difficult for traditional polymers, hence, such a popular method of using polymer blends. Numerous experiences concern the use of polymer blends, and both those based on traditional polymers and biopolymers indicate the possibility of using many methods of compatibility on a mass scale [5,6]. In the coming years, we can expect an increasing share of biopolymer blends in the market, which makes the topic of using methods of compatibility of this type of systems very actual, also in composite applications.

However, the current environmental policy, puts more and more emphasis on the acquisition of materials from renewable sources, thus, an increasing area of the market is occupied by bio-based materials, not necessarily biodegradable. One of the best example for that trend is the growing popularity of the biobased varieties of polyamides like PA11, PA610 [7,8]. It is worth adding that the characteristics of these materials do not differ in any way from their petrochemical counterparts like PA12 or PA6. In its present shape, the biopolymers production industry has ceased to focus on the production of cheaper varieties of biodegradable materials, but starts to replace the previously used petroleum products with their biobased varieties. This trend also affects the processing of polymer composites, since some of the epoxy resin systems can be actually partly or 100% biobased [9,10,11]. When natural fiber reinforcement is used, it is possible to obtain fully biobased composites. Unfortunately, due to the permanent cross-linking process of the epoxy resins, it will still not be an environmentally friendly material, thus, the modification of the already existing biopolymers still makes sense.

Currently one of the most popular biopolymer is poly(lactic acid) PLA, it is mainly used in the packaging industry as an ecological alternative to poly(ethylene terephthalate) PET [12,13,14]. Its popularity and a wide range of applications are due to fairly good mechanical properties, high modulus and strength, which are very similar to PET. Compared to other biopolymers, like thermoplastic starch materials, the processing stability is much better [15]. The weakness of the PLA resin is revealed when this material is used at elevated temperatures, usually above 50–60 °C. This temperature range corresponds to the glass transition temperature T_g_, above which the mobility of the polymer chains increases significantly, which leads to a softening of the material structure. PLA is a crystalline material, a natural solution to the problem of low thermal resistance is to increase the content of the crystalline phase in the material [16]. However, this is not a simple procedure, due to the slow crystallization process from the melt, especially for injection molding methods, where high cooling rates are the standard. The most effective way to improve the level of crystallinity is therefore to use heterogeneous nucleation. The most commonly used method is the addition of mineral fillers like talc particles [17,18,19,20], but there are more effective compounds that are now also used on an industrial scale, like potassium salts LAK or other nucleating agents [18,21,22,23]. It is noteworthy that even with the addition of the nucleating agents, the injection process parameters must be modified in terms of reducing the cooling speed, which means high mold temperature and long cooling time [18,24].

For composite materials processed by compression molding, the cooling rate is usually much lower than in the case of injection, and it is possible to obtain a high degree of crystallinity without the use of additional nucleation. In addition, when a sufficiently high reinforcement content is used, the growth of the crystalline phase on the surface of the fibers can lead to the formation of a more favorable spherulite morphology of the crystalline phase, which will improve the material reinforcing efficiency. Previous examples of research on PLA-based sandwich composites show a significant improvement in thermomechanical properties, sometimes without the need for nucleation or additional annealing. According to Huda et al. [25], the HDT of the 40% kenaf fiber reinforced composite can reach around 170 °C. Other studies reveal that the PLA/basalt fibers systems are also characterized by thermomechanical properties, while it was revealed that compaction temperature influence the properties [26].

The second major problem that arises during the use of PLA is its high brittleness, comparable to unmodified polystyrene. Low impact strength is a drawback of many polymers, hence very popular methods of improving these properties by using elastomers as impact modifiers [27]. The most frequently used modifiers are functionalized copolymers. The most common preparation process of this type of material involves reactive blending, which improves the compatibility of the elastomer phase with the polymer matrix. This kind of solution were used also for PLA-based materials, where EBA-GMA [28,29,30,31], EMA-GMA [32,33,34] or natural rubber [35,36,37,38] was used. Unfortunately, beside the effectiveness of this approach the addition of non-degradable petroleum-based cannot be consider as sustainable solution, which is the reason for development of new methods involving blending PLA with other elastomeric or soft biopolymers. So far, the different research studies confirmed the effectiveness of different types of biopolymers, like polycaprolactone—PCL [39,40,41,42,43], polyhydroxybutyrate—PHB [44,45,46,47,48], thermoplastic starch-TPS [49,50,51], poly(-3-hydroxybutyrate-co-3-hydroxyvalerate)—PHBV [52,53,54,55,56]. However, the most likely used biopolymer was PBAT, probably because of its popularity in foil production industry [2,57,58,59].

So far, the PLA/PBAT blend systems have been tested as materials intended for the production of films, injection molding or three-dimensional (3D) printing. The production of composite laminates based on PLA resin has not been the subject of intensive research, so far, but the appearance of this type of materials on the market indicates an interesting trend in the use of biopolymers The use of PLA as a matrix for composites and laminates was already investigated in case of ramie fibers [60,61], bamboo [62,63], cotton [64,65,66], kenaf [67] or flax [26,68,69,70]. The use of flax fiber (FF) reinforcement was for us the most promising concept, since the reinforcing efficiency for already investigated PLA/FF composite systems was very high, which was confirmed in many studies [68,71,72,73,74].

In the presented study, several types of PLA-PBAT based composite samples were prepared. Flax fiber fabric was used as reinforcement, while the laminates were manufactured using compression molding technique. The reference samples were prepared using injection molding technique. In order to increase the composition compatibility part of the samples was prepared using reactive extrusion process. For this purpose we used the epoxy-based chain extender additive. Given that the main goal of the research was to improve the mechanical properties of composites, all samples were tested in this respect. Additionally, the differential scanning calorimetry (DSC) thermal analysis measurement were conducted, while the main purpose of these tests was to evaluate the PLA crystallinity level. Thermo-mechanical properties were evaluated using dynamic mechanical thermal analysis (DMTA) measurements and heat deflection temperature (HDT) comparison. The research is also supplemented with structure observations, which were prepared using scanning electron microscopy method (SEM). During the analysis, the main emphasis of the research is focused on identifying the structural differences resulting from the use of the PLA-PBAT blends instead of pure PLA. Due to the different thermal condition of injection molding and compression molding process, the results analysis takes into account the results difference between these two techniques.

## 2. Materials and Methods

### 2.1. Materials

Two types of polymer resins were used during the investigation. The first one was poly(lactic) PLA Ingeo 3251D from NatureWorks (Minnetonka, MN, USA), MFI = 30–40 g/10 min (190 °C, 2.16 kg). This type of PLA is intended for injection molding applications. The second polymer type was polybutylene adipate terephthalate PBAT foil grade Ecoflex C1200 type from BASF (Ludwigshafen, Germany), MFI = 2.7–4.5 g/10 min (190 °C, 2.16 kg). For the purpose of reactive extrusion process we used the epoxy functionalized chain extender (CE), in our case it was random styrene-acrylonitrile-glycidyl methacrylate terpolymer type SAG-008 from the company Fine-Blend (Shanghai, China), material was supplied by DSKH GmbH. Composite reinforcement consists of flax fiber fabric, we used the natural flax. The density of the used plain weave fabric was 260 g/m^2^.

### 2.2. Sample Preparation

The first stage of the materials preparation consists of melt blending process. The extrusion process was performed using twin-screw extruder type EH-16.2D from Zamak Mercator (Skawina, Poland). Before extrusion, the PLA and PBAT pellets were dried using cabinet dryer for 12 h at 70 °C to avoid the presence of moisture, the CE pellets were used as received. During extrusion the temperature profile was the same for all blends (190 die–190–190–180–180–170–170–160–150 °C). The screw speed was set to 100 rpm, while the extrusion rate was 4 kg/h. The prepared extrudate was cooled and pelletized. The reference samples were using injection molding technique. For this purpose we used Engel E-mac50 machine (Engel GmbH, Schwertberg, Austria). Before molding pellets were dried again (12 h, 70 °C). The necessary dumbbell specimens were prepared using following conditions: injection temperature—190 °C, mold temperature—40 °C, injection speed—100 cm/s, injection/holding pressure—1050/850 bar, holding/cooling time—10/40 s. During the preparation stage 6 types of PLA/PBAT blends was melt mixed. The first group of materials contained 10%, 20% and 30% of the PBAT resin, while a second analogous group of samples was prepared with the addition of CE modifier. The last type of prepared samples was the reference PLA. Sample markings indicate PBAT content and possible presence of CE compound.

Composite samples were prepared using film stacking method. At the first stage we prepared a set of polymer foils. For this purpose, a small amount of polymer pellets was placed between two PTFE foils, after that, the material was placed between hydraulic press tables. The press was heated to 190 °C, after 1 min of preheating the 20 MPa pressure was applied for 30 s. The thickness of a single foil was around 0.2 mm. After that the obtained film was cooled in air. Composites samples were prepared by stacking 6 layers of flax fabric and 5 films. The press temperature was again 190 °C, while the preheating time was 3 min. The maximum pressure of 20 MPa was applied for another 3 min, while after that time the cooling stage was started. Samples were demolded after around 15 min, at 40 °C. Before compression molding, both films and fabrics were dried. In case of polymer films procedure takes 6 h (at 70 °C), while for flax fabric (FF) we used 24 h (at 100 °C). The list of materials prepared during the extrusion stage is presented in the Table 1.

### 2.3. Material Characterization

Thermal properties of the prepared samples were evaluated using differential scanning calorimetry method (DSC). For this purpose small size samples (≈ 5 mg) were cut from the original specimens and placed inside the aluminum crucible. The measurement program consists of standard heating/cooling/heating procedure was performed from 20 °C to 220 °C with the heating/cooling rate of 10 °C/min. All measurements were conducted under protective atmosphere of the nitrogen. Phoenix 204 F1 DSC apparatus from Netsch company (Selb, Germany) was used during the tests. The crystallinity of the samples was calculated using the following Equation (1):

(1) $$\%\;\mathrm{Crystallinity}=\chi_{c}=\;100\;\times \;\frac{\Delta H_{m}-\;\Delta H_{c}}{\Delta H_{m}^{0}}$$

where $\Delta H_{m}$ is the measured melting enthalpy, $\Delta H_{c}$ is the measured enthalpy of cold crystallization, and $\Delta H_{m}^{0}$ is the theoretical melting enthalpy of 100% crystalline PLA was taken from the literature to be 93 J/g [75,76].

Viscoelastic properties were analyzed using DMTA methods. A test were performed using rectangular samples, with dimensions 50 mm × 10 mm × 3 mm. The test temperature range was set to 25–150 °C, while the heating rate was 2 °C/min. The deformation strain was 0.01% and the frequency 1 Hz. For the purpose of the study, we used MCR 301 apparatus from Anton Paar (Graz, Austria), the machine was equipped with solid rectangular sample fixture, while the tests were performed using torsion mode. DMTA measurements were supplemented with heat deflection temperature (HDT) thermo-mechanical properties tests. The measurements were conducted using HDT/Vicat RV300C oil bath apparatus form Testlab (Warsaw, Poland). Measurements were performed, according to ISO 75 standard [77] using standard samples. The heating rate was set to 2 °C/ min, while the applied load was 0.45 MPa.

Mechanical tests were performed using the Zwick/Roell Z010 machine (Zwick/Roell, Ulm, Germany), tests were performed using tensile and flexural type of measurements. Tensile tests were conducted according to ISO 527 standard [78], where the gauge length was 100 mm and the crosshead speed was set 5 mm/min. Flexural tests were conducted according to ISO 178 standard [79], where the span distance was 48 mm, the test speed was 2 mm/min. Mechanical tests are complemented by impact resistance measurements, we used the notched Charpy method. Since the tests were performed according to ISO 179 standard [80] the specimen notch depth was 2 mm. The used equipment was Zwick/Roell HIT25 hammer, attached with 5 J energy pendulum. Mechanical properties evaluation was supplemented with falling weight tests, where we used the tubular impact tester. The apparatus was equipped with 1 kg weight, the impactor diameter was 20 mm, while the die diameter was 27 mm. The impact energy of 5 N was applied during the tests.

Structure observations were carried out using the scanning electron microscopy method (SEM). Pictures were captured using EVO 40 SEM microscope from Karl Zeiss (Jena, Germany). The observed surface was obtained from the fractured sample side, before scanning specimens were sputter coated with thin layer of gold.

## 3. Results and Discussion

### 3.1. Differential Scanning Calorimetry—Phase Transition and Crystallinity

**Figure 1:**
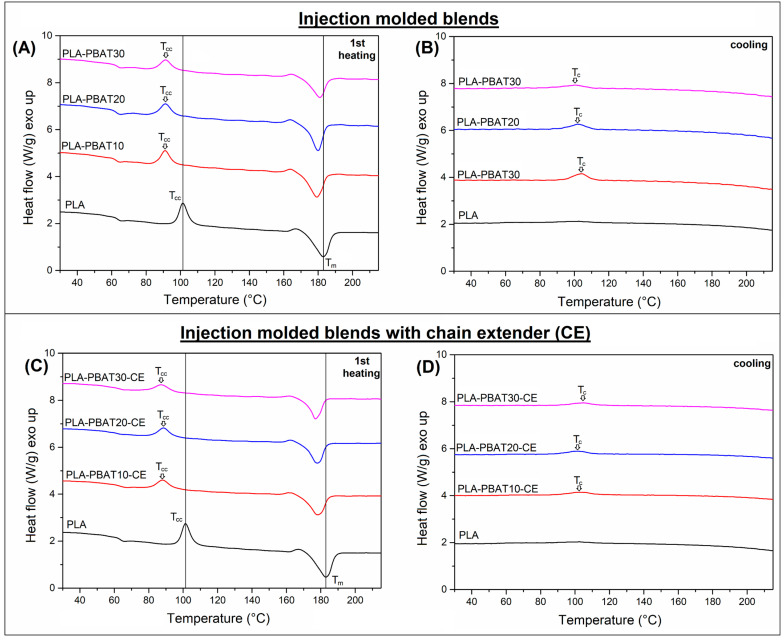
DSC thermograms for injection molded samples. The 1st heating and cooling DSC plots for (**A**,**B**) PLA-PBAT blends and (**C**,**D**) PLA-PBAT-CE materials.

**Figure 2:**
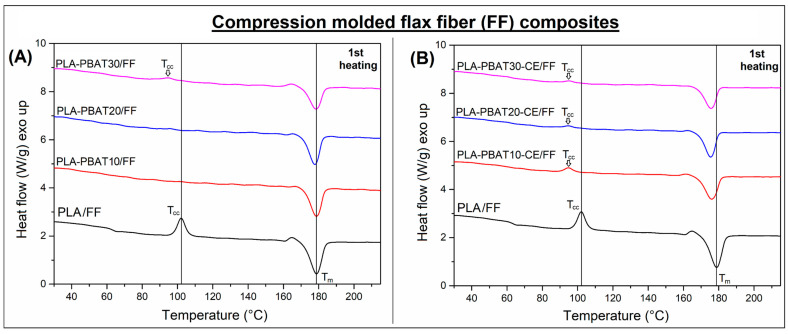
DSC thermograms for compression molded composites. The first heating thermograms for (**A**) unmodified PLA-PBAT/FF materials and (**B**) similar composites prepared with addition of CE.

The results of the DSC scans for the injection molded samples can be seen in the Figure 1, while Figure 2 presents the thermograms for the FF reinforced composites. The basic thermal properties obtained for injection molded and compression molded samples are collected in Table 2, including the calculated crystallinity of the PLA phase. As can be predicted, the appearance of the DSC plots for injection molded and compression molded samples is different, it is especially visible analyzing the first heating thermograms. It is worth adding that, in the case of compression molded samples, measurements were performed on fragments that did not contain flax fibers so as to eliminate the problem of their variable content in small DSC samples.

The 1st heating thermograms for all injection molded samples reveals the presence of the cold crystallization phenomenon, which suggest that the PLA structure was highly amorphous after the sample demolding. This kind of behavior is required for most of the thermoplastic polyesters, also for PLA, since it helps in maintaining the transparent structure of the material, especially during processing of packaging items like foils or food containers. However, when it comes to the processing of technical goods, the low crystallization speed hinders the formation of more thermal resistant crystalline phase. For the prepared blends it can be seen that the position of the cold crystallization peak is shifter to lower temperature for all types of PLA/PBAT blends. The second important feature that distinguishes the appearance of blend thermograms from pure PLA is the presence of small exothermic peak during the crystallization stage. This kind of changes suggest the nucleation ability of the PBAT phase domains, which can be considered as favorable feature. The positive aspects of the nucleation of the PLA phase structure is revealed during the analysis of composite samples (Figure 2), where the plots or of the first heating revealed that the scale of the cold crystallization phenomenon is largely limited. For PLA/PBAT10 and PLA/PBAT20 sample the exothermic peak was not recorder, while for PLA/PBAT30 blend the area under the peak was only 5.5 J/g, while the value recorded for pure PLA was 22 J/g. Interestingly, for CE modified samples the presence of cold crystallization phenomenon was more visible, however, again the peak area for none of the sample exceed 6 J/g. The consequence of the described phenomena is an increase in the crystallinity of the PLA phase, which obviously translates into an increase in thermomechanical properties.

In the case of PLA-based mixtures, the phenomenon of nucleation of the crystal structure by inclusions of the dispersed phase of the mixture is common, also for blends containing PBAT. However, it is worth mentioning that the scale of these changes, although visible, is relatively small and has little effect on the mechanical and thermomechanical properties of the injection molded samples. Despite the fact that for the tested samples the increase in crystallinity was sometimes even more than 40%, from 23% for pure PLA to over 30% for selected blends. However, the phenomenon of cold crystallization still occurs for all injected molded specimens, which clearly proves that for samples of this type the content of the amorphous phase is still predominant. As the calculations show, the increase in the content of the PLA crystalline phase for the prepared composite samples is mainly related to more favorable processing conditions, primarily slow cooling rate. Even for pure PLA the increase of the crystalline phase content was large, from 23% (injection molding) to around 41% in composites. The highest values, above 60%, occur for samples with the addition of 10% and 20% of PBAT. With the addition of 30% PBAT, the increase was reduced and the content of the PLA crystalline phase was reduced to 54%, which would suggest that a significant content of the dispersed phase may limit the growth of the crystallites, which may be caused by the bonding of some PLA chains at the PLA-PBAT phase interface. An important confirmation of this assumption is the appearance of a cold crystallization peak for PLA-PBAT30 samples.

Even more visible limitation of the PLA crystal phase growth was observed for the samples with CE addition. For all samples subjected to reactive extrusion the crystallinity was reduced below 60%. Moreover, for all specimens, the presence of pronounced cold crystallization peak was detected. This type behavior can be considered confirmation that a significant amount of polymer chains have been bound at the PLA-PBAT interface. The presented results confirm the effectiveness of the used strategy of phase compatibilization.

### 3.2. Thermo—Mechanical Analysis—Heat Resistance

**Figure 3:**
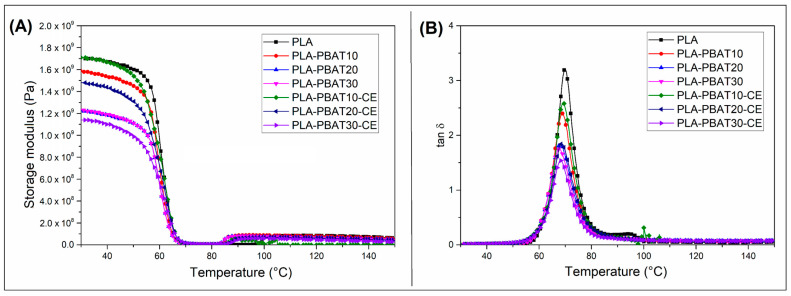
DMTA analysis results for injection molded blends. (**A**) storage modulus curves, and (**B**) tan δ results.

**Figure 4:**
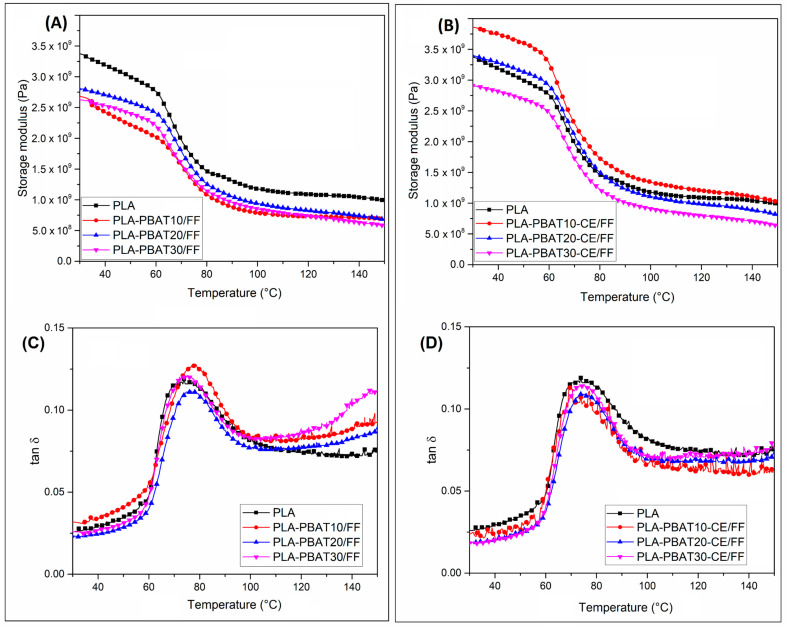
DMTA analysis results for compression molded FF composites. Storage modulus and tan δ curves for (**A**,**C**) PLA-PBAT matrix type composites, and (**B**,**D**) PLA-PBAT-CE matrix composites.

Viscoelastic properties of the blends and composites samples are presented in the Figure 3 and Figure 4, where storage modulus and tan δ curves are plotted. For all plots the curves for blends are compared with the pure PLA sample. As can be predicted for injection molded samples the stiffness is highly dependent on the PBAT phase content (see Figure 3), since the lowest storage modulus values are reported for PLA-PBAT30-CE samples. The effect of the addition of the PBAT phase is also clearly visible in the tan δ plots, where the area under the curve peak at T_g_ is the lowest for PBAT rich samples.

The storage modulus comparison form the Figure 4A reveals that the stiffness of the PLA/PBAT composites was lower than for the pure PLA sample. Interestingly, there is no clear relationship between the content of the PBAT phase and the storage modulus values, which might be caused by few factors. The first factor might be related to some differences in the PLA crystallinity, which may be due to differences in the rate of the sample cooling. The second factor may be related to the possible differences in the reinforcement content for the tested sample. Apart from some inaccuracies, a significant change in relation to the injection-molded sample is the appearance of the storage modulus curve itself. The typical sharp drop of the PLA storage modulus around the T_g_ range is not apparent for the prepared composite samples, which confirms the significant increase in the crystalline phase content.

Considering the thermomechanical properties, this kind of changes is very beneficial and improves the heat resistance. Interestingly, for CE modified samples, the storage modulus values were even improved. For compatibilized samples, containing 10% and 20% of PBAT phase, the storage modulus values were even higher than for pure PLA-based composites. The occurring correlations clearly indicate that in the case of materials with the CE addition, the reinforcement efficiency is higher. Taking into account the results obtained for the injection molded specimen it seems that the main reason for that improvement might be related to the presence of effective reaction between the end groups of PLA and PBAT chains and the hydroxyl groups occurring on the surface of the flax fiber. Similar behavior was observed for other composite systems modified with epoxy functionalized modifiers, like PLA/sisal [81,82,83], or PLA/silk [84].

Usually, the increase in the effectiveness of composite reinforcement can also be con-firmed by analyzing of the tan δ. However, for the described case the differences between the curves for PLA/PBAT composite samples are very negligible and rather random. More noteworthy is the change in the size of the tan δ peak at T_g_ transition, where the reduction in relation to injection molded samples is very significant. With such low values of this factor, it is difficult to find clear correlations of the PBAT content or the addition of CE, hence, the analysis of any change in the value or temperature shift of the tan δ peak can be treated as negligible.

The large efficiency of the flax fabric reinforcing effect together with the significant improvement in PLA crystalline phase content are leading to the increase of the thermomechanical performance of the laminated samples. For the purpose of our study, the HDT test were conducted (see Table 3). As can be predicted after blending with PBAT the thermal resistance of the injection molded samples was not improved. However, what is noteworthy, even for PLA/PBAT30 samples, there was no visible decrease in the HDT temperature and for most of the samples the test results did not differ more than 2 °C from the reference PLA sample. The situation changes significantly for composite samples, where the heat resistance was strongly improved. The highest HDT was recorded for pure PLA composite where the temperature reach 145 °C. The addition of PBAT results in visible reduction of the deflection temperature, both for composites prepared from the pure blends and CE modified compositions. However, even for the worst result observed for PLA/PBAT 30 samples, the HDT of 98 °C is still about 40 °C higher than the result for the injection molded samples. Unfortunately, the validation of the obtained results is quite a difficult issue, while in the previous research for PLA-based laminates, the authors rarely referred to the thermomechanical properties like HDT or Vicat, sometimes indicating low values (≈ 60 °C) similar to amorphous PLA varieties [85]. Other studies showing the thermal resistance above 100 °C, and suggest a significant influence of the fiber content [71,86]. It does not change the fact that our research clearly suggests that the PBAT addition causes visible decrease of the composite heat resistance, but the scale of the reduction is still acceptable and the obtained properties are very good.

### 3.3. Mechanical Properties Characterization

**Figure 5:**
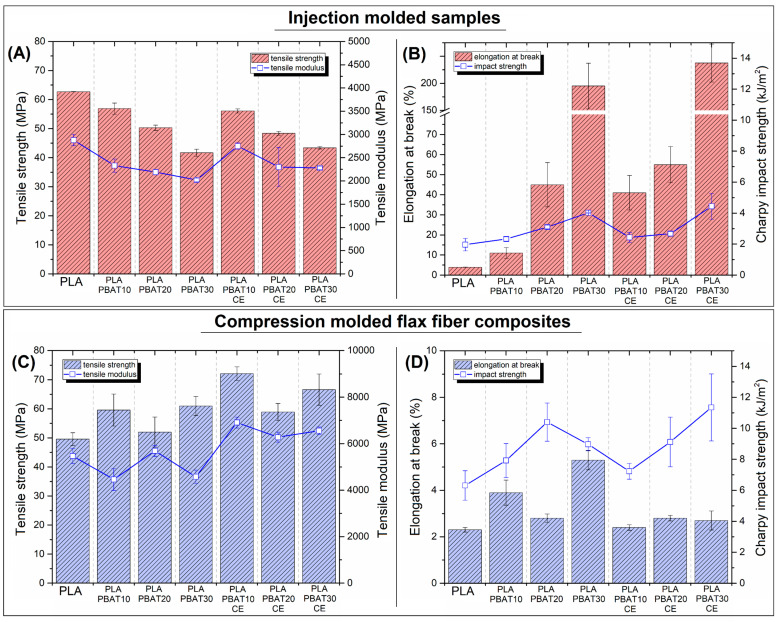
The comparison of the mechanical properties for (**A**,**B**) injection molded blends, and (**C**,**D**) compression molded FF composites. The plots are presenting the results of tensile strength, tensile modulus, elongation at break and Charpy notched impact strength.

The already presented results confirmed that for the prepared composites the thermal and thermomechanical properties are highly improved. This section is presenting the results of the mechanical properties investigation. The full list of properties obtained during the tensile, flexural and impact resistance measurements are collected in the Table S1 (Supporting Information section), while the stress/strain curves are collected in the Figure S1. The results in the form of graphical plots (Figure 5) are presenting the tensile modulus, tensile strength, elongation at break and notched Charpy impact strength.

The plots presented in the Figure 5A,B reveal some obvious trends regarding the injection molding samples. It is clear that both tensile strength and modulus are decreasing with the addition of PBAT phase. It is noteworthy that there is no difference between the standard blends and the materials prepared with reactive extrusion process. The decrease in tensile modulus and strength for PLA-PBAT30 samples is 30%, and 33%, respectively, in relation to the PLA sample. Therefore, it should be noted that the initial properties of the blends are strongly deteriorated. More favorable changes were recorded for elongation at break values, where for PLA-PBAT30 and PLA-PBAT30-CE samples the maximum strain was 195%, and 237%, respectively. Improvement was also observed for the values of impact resistance, where again for the samples containing 30% PBAT the impact strength was slightly higher than 4 kJ/m^2^, while for reference PLA it was below 2 kJ/m^2^. The increase in sample toughness is significant, however, in the case of other types of PLA/PBAT blend systems, much better results were achieved. In the case of the presented research it is clear that the values obtained for the injection specimens are only a benchmark for the results for the composite samples.

The results for the composite samples are presented in the Figure 5C,D. After addition of the flax fiber reinforcement the tensile modulus for PLA/FF samples doubled comparing to injection molded sample. Interestingly, for PBAT-CE modified samples the stiffness was even higher, however, any dominant trend of these changes cannot be identified, while the highest stiffness is noted for PLA-PBAT10-CE samples. The increase in tensile strength for composite samples is not so obvious, because for the PLA/FF sample strength was even lower than for injection molded samples, 49.6 MPa, to 62.7 MPa, respectively. For samples with the addition of PBAT, a slight increase in strength values is visible, however, also here there is no clear trend of changes. Due to the introduction of the FF reinforcement, the elongation at break values were strongly reduced, comparing to injection molded samples. The strain values for most of the composites was close to 2%, while slightly higher values for PLA-PBAT10 and PLA-PBAT30 cannot be interpreted as indicative of a property change. More important results can be observed for the impact strength values, where even the results for PLA/FF samples were three times higher than the reference PLA. The addition of PBAT also improves this result, while for PLA-PBAT30-CE sample the impact strength was around 11 kJ/m^2^.

**Figure 6:**
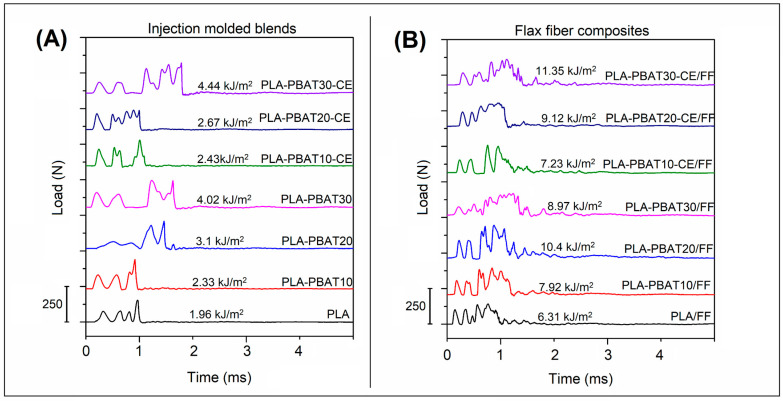
The load/time curves for the (**A**) injection molded samples, and (**B**) flax fiber reinforced laminates.

During the impact measurements, we used a hammer equipped with a force sensor, which allowed us to determine load curves over time. The load/time plots are presented in the Figure 6. The plot analysis clearly show that in the case of the PBAT modified samples, the deformation time significantly increases, which leads to a higher energy consumption during the process of the sample crack. For the composite samples, the crack propagation was even longer, while the shape of the curve was more complex, which suggests the presence of several overlapping crack propagation mechanisms, like fiber pull-out, fiber breaking and interface debonding.

**Figure 7:**
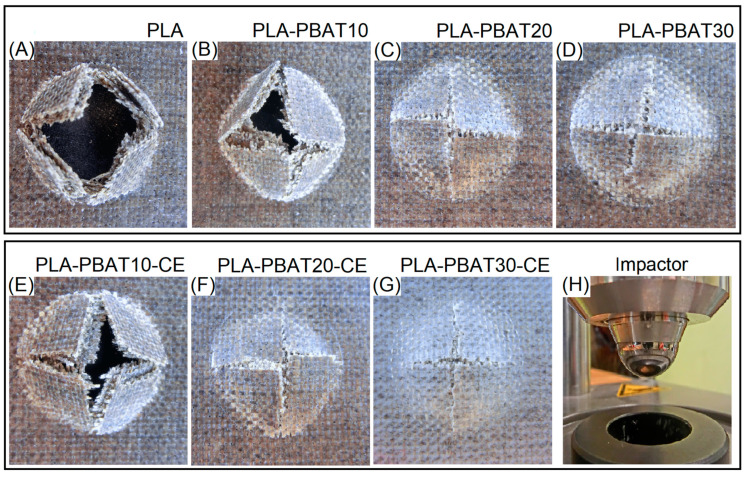
The results of the falling weight impact measurements for (**A**) pure PLA/FF laminate, (**B**–**D**) PLA-PBAT matrix samples, (**E**–**G**) PLA-PBAT-CE matrix composites, (**H**) the appearance of the apparatus impactor.

The impact resistance was also investigated using the falling weight method, and in our case, the tests were performed using simple tubular apparatus attached with spherical impactor. The results for tests carried out with a load of 5 N are presented in the Figure 7, the shape of the device is also revealed. The results of the tests are presented in the form of fracture appearance comparison. For pure PLA samples, where the impactor penetrate the whole thickness of the sample, the individual layers of the fabric were delaminated. This indicates a clear lack of adhesion at the matrix/fiber interface, these results were also confirmed during Charpy measurements. Some minor changes are reported for 10% PBAT samples where the sample fracture reveals that the delamination of the composite layers was limited. The dominating deformation mechanism was more related to fiber breakage. However, still the composite structure was perforated. The size of the crack was limited for PBAT rich specimens. For PBAT 20% and 30% the laminate structure was not penetrated, only the outer layer of the laminate cracked. The difference between the crack size are slight. However, the PLA-PBAT30-CE/FF sample is clearly characterized by the smallest fracture.

Summarizing the mechanical test analysis is can be state that the addition of PBAT phase into the PLA matrix increase the mechanical properties of flax fiber reinforced composites, especially for materials prepared by reactive extrusion process. Both tensile modulus and strength are slightly improved, but the most favorable changes are observed for the impact resistance tests. For both Charpy measurements and falling weight tests the best results were obtained for PLA-PBAT30-CE/FF samples.

The mechanical performance of the composite samples can be evaluated by separate analysis of the particular properties like tensile strength, modulus or impact strength. For many reasons this approach is not optimal, since it requires the specialized knowledge and is time consuming. For many reasons, the use of multifactorial performance factors is a more efficient way for properties evaluation. One example for this approach is the use of specific strength/modulus comparison, where beside the actual value of the measured coefficient, the sample density is also taken into account [87,88]. Since, in our study, only one type of reinforcing fabric at constant amount was used, the density differences will be negligible. This is why we used other multifunctional factors, in our case, reinforcing efficiency C-factor and brittleness B-factor. C-factor values are calculated from the storage modulus values at two different temperatures, and in our case, it was 25 °C and 100 °C, which temperatures are below and above the glass transition. For the purpose of our study we calculate the C-factor vales according to following Equation (2):

(2) $$C=\frac{{\left(E_{g}^{\prime }/E_{r}^{\prime }\right)}_{composite}}{{\left(E_{g}^{\prime }/E_{r}^{\prime }\right)}_{matrix}}$$

where the $E_{g}^{\prime }$ and $E_{r}^{\prime }$ storage modulus values are collected from thermogram in glassy (25 °C) and rubbery (100 °C) region. In our study, C-factor evaluation is helpful in assessing the reinforcement efficiency for different types of composite matrix. A lower value of the coefficient translates into a higher effectiveness of the used reinforcement, thus a positive effect of the applied compatibility method.

For brittleness factor calculation the necessary numerical values are taken from the DMTA analysis and static tensile test. Storage modulus E’ (at room temperature = 25 °C) and elongation at break $\varepsilon_{b}$ results are used to calculate the brittleness factor according to the following Equation (3):

(3) $$B=1/\left(\varepsilon_{b}\cdot E^{\prime }\right)$$

**Figure 8:**
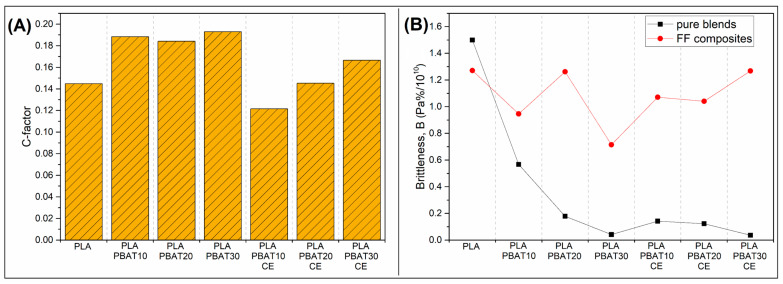
The comparison of (**A**) the C-factor values for FF reinforced composites, and (**B**) Brittleness coefficient values for injection molded blends and FF composites.

The first view at the C-factor calculation results (Figure 8A) reveals that highest reinforcement efficiency was obtained for PLA-PBAT10-CE samples. The gradual introduction of PBAT leads to the increase of the C-factor values, which translates into deterioration of the material performance. It is worth noting that the value of the coefficient for samples without CE addition are the highest, which suggest the love reinforcing efficiency. Interestingly, for all PLA-PBAT samples the C-factor values are very similar. The comparison of these two groups of materials clearly shows the positive effect of the use of the reactive compatibility method, which clearly indicate the appearance of strong interactions at the fiber-matrix interface. The low C-factor values for the pure PLA samples might suggest that, even without the compatibilization the fiber-matrix interface, adhesion for PLA/FF composite was very strong. This conclusion is not entirely true, because in the discussed case, the modification of the matrix was associated with a significant reduction in stiffness, which translates into the final result of the C-factor value. Therefore, in the presented analysis, the result for the unmodified PLA sample indicate high efficiency of reinforcement, but they cannot be compared to other blend-based samples.

The brittleness factor values are collected in the Figure 8B. where the results for injection molded samples and FF composites are compared. Since the modification of the injection molding samples is leading to large improvement in elongation at break values, the lowest brittleness was observed for samples containing 30% PBAT in the structure. The results for PLA/PBAT30 and PLA-PBAT30-CE samples are very close, but for samples with addition of 10% and 20% PBAT, the advantage of the reactive extrusion process becomes apparent. The results obtained for the injection-molded samples confirm the high efficiency of the PBAT additive in improving the brittleness index of the matrix. The results of for the FF reinforced materials revealed the large improvement in brittleness, which can be consider as unfavorable. However, the B factor values are lower than the results for pure injection molded PLA. That kind of behavior suggest that the initial brittleness of the PLA samples was very high, which was confirmed by impact resistance studies. The addition of flax fiber reinforcement leads to visible improvement, however the lowest brittleness was observed for PBAT rich injection molded samples.

### 3.4. Composite Structure—Scanning Electron Microscopy Analysis

**Figure 9:**
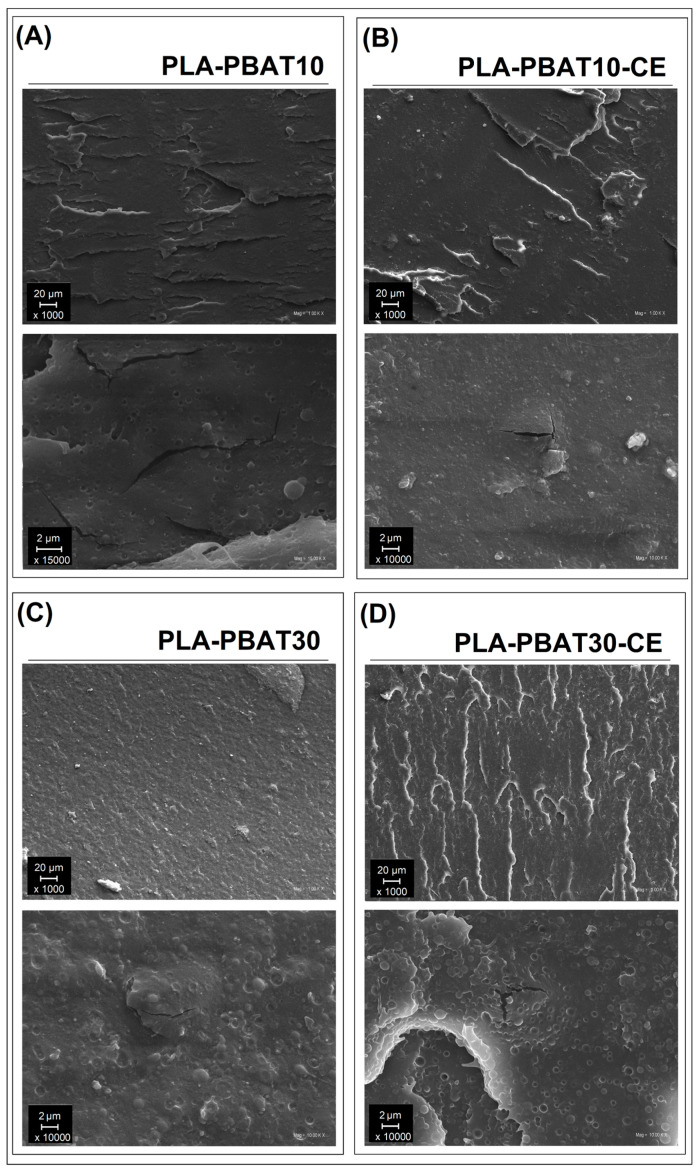
SEM picture presenting the fractured surface of the impact test specimens for samples containing 10% and 30% of the PBAT phase. (**A**) PLA-PBAT10, (**B**) PLA-PBAT10-CE, (**C**) PLA-PBAT30, (**D**) PLA-PBAT30-CE.

**Figure 10:**
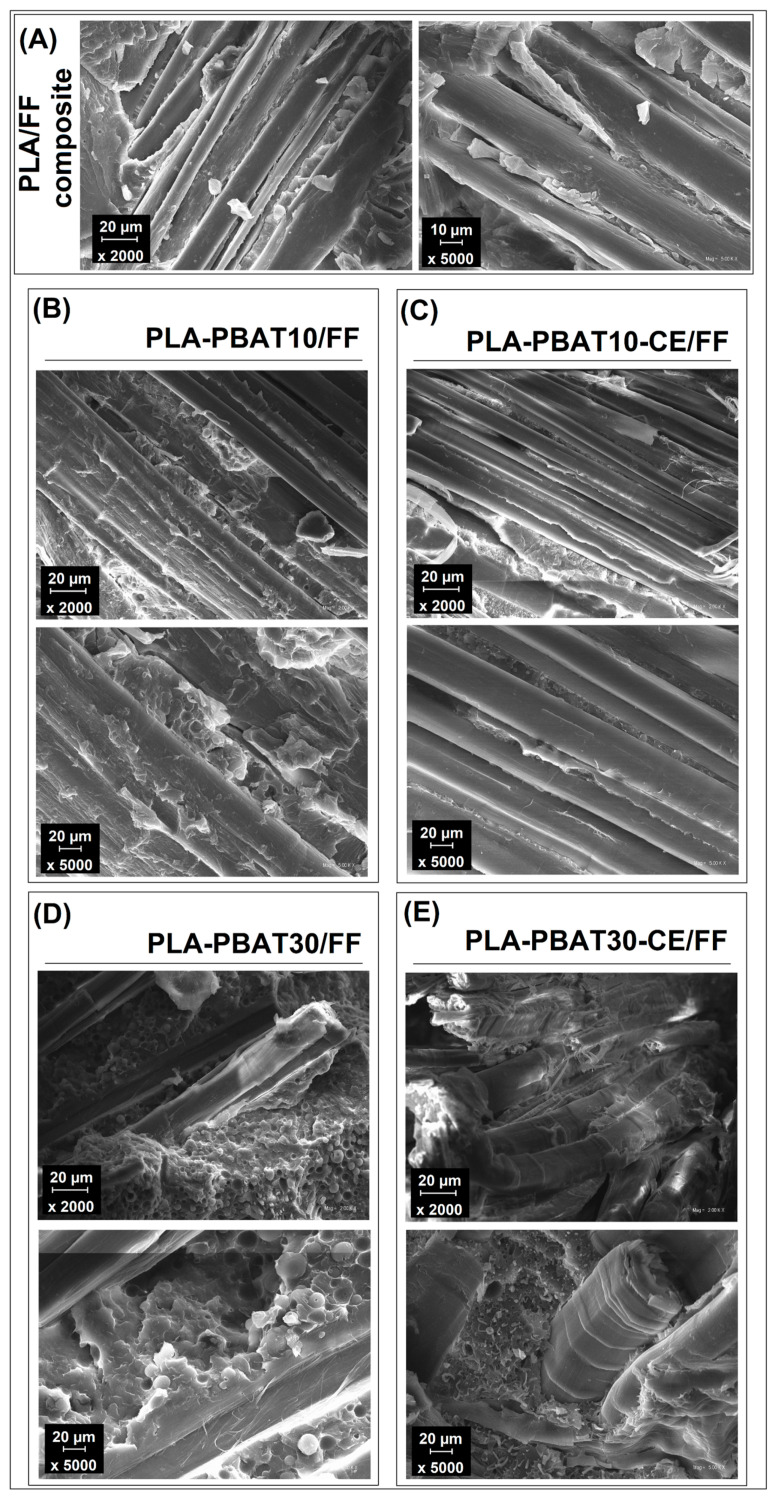
Structure of the FF reinforced composites. (**A**) PLA/FF samples, (**B**) PLA-PBAT10/FF, (**C**) PLA-PBAT10-CE/FF, (**D**) PLA-PBAT30/FF, (**E**) PLA-PBAT30-CE/FF.

The SEM analysis was performed for both injection molded blends (Figure 9) and compression molded laminates (Figure 10). The specimens were obtained from the fractured surface after the Charpy impact tests. The initial analysis for the unreinforced PLA-PBAT blends reveals the brittle fracture characteristic, but there are some differences in surface appearance between samples containing 10% and 30% of the PBAT. Since the difference is limited only to the surface roughness, it is difficult to indicate significant differences in the fracture mechanism. A more detailed view shows the two phase structure of the blends. The size of the PBAT inclusions are below 1 µm, while still the droplet morphology is spherical, which means that the dominating fracture micro-mechanism was interface debonding. The structure observations are confirmed by the impact tests results, where the impact resistance for PLA-PBAT materials was higher than for pure PLA. However, for other studies where plastic deformation mechanism was revealed, the impact resistance was improved several time [89].

The appearance of the composite structure presented in the Figure 10 is dominated by the presence of the flax fibers. However, the large amount of fibers still does not interfere with the observation of the matrix structure, since the content of the reinforcing fabric was around 50 wt %. The structure of PLA/FF sample can be seen in the Figure 10A. It is clear that the more brittle characteristic of this materials was connected with poor interfacial interactions. Large gaps between the fiber surface and PLA matrix reveals the lack of strong adhesion. These features, together with PLA brittleness, make the properties of PLA/FF laminate relatively low. The addition of 10% PBAT into the matrix structure reveals some changes in the fracture behavior. The main difference refers to the limited number of the matrix cracks, which confirmed the higher ductility of the PLA-PBAT blends. The presence of visible gaps on the matrix-fiber interface confirmed again the poor adhesion level. More favorable structural changes are observed for PBAT rich samples. For PLA-PBAT30/FF sample the large inclusion of PBAT phase can be distinguished between the fibers. Higher magnification allows to confirm the presence better adhesion on the fiber surface, while there was no gap at the composite interface. For PLA-PBAT-CE/FF sample the matrix structure appearance confirmed the presence of plastics deformation of the PBAT inclusion. The structure fibrillation phenomenon leads to higher energy consumption during the crack propagation [90], which finally improves the Charpy impact strength and falling weight test results.

## 4. Conclusions

The results of the presented study confirmed that the use of PLA/PBAT matrix system for the preparation of flax fiber reinforced composites was a favorable strategy. Compared to pure PLA-based composites, the impact properties of the modified composites significantly improved, especially for samples prepared with the addition of the chain extender. Since the difference in notched Charpy impact strength between the pure PLA (1.96 kJ/m^2^) and PLA-PBAT30-CE blend (4.44 kJ/m^2^) reached around 225%, the use of the flexible PBAT as a toughening agent can be considered as a successful strategy for laminated composite preparation. The increase in impact resistance for the polymer matrix translated into better results for composites, while improvement reached about 180%.

Noteworthy is the use of the reactive compatibilization procedure, which also improves the tensile modulus and strength, which behavior partly confirmed the presence of enhanced adhesion at the matrix-fiber interphase. Interestingly, the presence of PBAT phase inclusions increase the crystallinity of the PLA phase. For example, the tensile modulus for PLA-PBAT10-CE/FF composite was 6910 MPa, while is around 25% higher than reference PLA/FF material.

Unfortunately, for injection molded samples the HDT results were similar for all samples, while for laminated composites, the presence of soft PBAT phase leads to deterioration of the heat resistance.

The results of the presented study confirmed the effectiveness of the developed concept of PLA composite toughening. However, the overall performance of the composite samples was not satisfactory compared to the standards of epoxy/glass fiber based laminates. Since the mechanical properties of the natural fibers are not competitive compared to synthetic fibers, the planned research study will be focused on the development of hybrid types of materials reinforced with natural/synthetic type of reinforcement.

## Figures and Tables

**Table 1:** The list of the prepared blends with formulations.

Sample	PLA	PBAT	CE
	(%)	(%)	(phr)
PLA	100	-	-
PLA-PBAT10	90	10	-
PLA-PBAT20	80	20	-
PLA-PBAT30	70	30	-
PLA-PBAT10-CE	90	10	0.5
PLA-PBAT20-CE	80	20	0.5
PLA-PBAT30-CE	70	30	0.5

**Table 2:** The basic thermal properties for injection molded and compression molded samples.

Matrix Type	Injection Molding			Compression Molding		
	Δ*H_cc_* (J/g)	Δ*H_m_* (J/g)	χ*_c_* (%)	Δ*H_cc_* (J/g)	Δ*H_m_* (J/g)	χ*_c_* (%)
PLA	36.9	58.5	23.0	22.8	61.6	41.4
PLA-PBAT10	23.9	50.6	31.6	-	51.3	60.8
PLA-PBAT20	25.3	50.4	33.5	-	50.7	67.6
PLA-PBAT30	21.6	40.9	29.4	5.5	41.0	54.1
PLA-PBAT10-CE	22.1	47.0	29.5	5.8	45.9	47.5
PLA-PBAT20-CE	20.3	42.0	28.9	4.6	42.4	50.4
PLA-PBAT30-CE	18.2	39.4	32.3	3.8	40.5	55.9

**Table 3:** Results of the heat deflection temperature (HDT) measurements

Matrix Polymer	HDT (0.45 MPa) (°C)	
	Injection Molded Blends	Flax Fiber Composites
PLA	60.5 (±0.4)	145.4 (±4.8)
PLA-PBAT10	61.9 (±1.2)	131.0 (±5.5)
PLA-PBAT20	62.2 (±0.5)	112.4 (±0.4)
PLA-PBAT30	59.1 (±0.3)	98.4 (±8.3)
PLA-PBAT10-CE	58.1 (±0.8)	126.6 (±1.6)
PLA-PBAT20-CE	59.8 (±0.7)	110.1 (±4.7)
PLA-PBAT30-CE	61.3 (±0.8)	109.0 (±0.2)

## Data Availability

Data is contained within the article or supplementary material.

## Supplementary Materials

The following are available online at https://www.mdpi.com/1996-1944/14/6/1523/s1, Table S1: The full list of mechanical properties obtained from the tensile, flexural and Charpy impact measurements, Figure S1: The macroscopic appearance of the 3D printed samples, pictures are presenting the impact test specimens after the fracture.
