# Supplementary material for: Development of Toughened Flax Fiber Reinforced Composites. Modification of Poly(lactic acid)/Poly(butylene adipate-co-terephthalate) Blends by Reactive Extrusion Process
Source: Materials (Basel). 2021 Mar 20;14(6):1523. doi: 10.3390/ma14061523 (PMC8003650; doi:10.3390/ma14061523)
Supplement: Supplementary file 1 [file materials-14-01523-s001.pdf]

materials

SUPPLEMENTARY

Development of Toughened Flax Fiber Reinforced Composites. Modification of Poly(Lactic Acid)/Poly(Butylene Adipate-Co-terephthalate) Blends by Reactive Extrusion Process

Jacek Andrzejewski [1,2,*] and Michał Nowakowski [2,3]

[1] Polymer Processing Division. Institute of Materials Technology, Faculty of Mechanical Engineering, Poznan University of Technology, ul. Piotrowo 3, 61-138 Poznan, Poland
[2] MATRIX Students Club, Polymer Processing Division, Poznan University of Technology, ul. Piotrowo 3, 61-138 Poznan, Poland; michal.m.nowakowski@student.put.poznan.pl
[3] Faculty of Materials Engineering and Technical Physics, Poznan University of Technology, ul. Piotrowo 3, 60-965 Poznan, Poland
* Correspondence: jacek.andrzejewski@put.poznan.pl; Tel.: +48 616475858

Citation: Andrzejewski, J.; Michał Nowakowsk Development of Toughened Flax Fiber Reinforced Composites. Modification of Poly(Lactic Acid)/Poly(Butylene Adipate-Co-terephthalate) Blends by Reactive Extrusion Process. *Materials* **2021**, *14*, 1523. https://doi.org/10.3390/ma14061523

Academic Editor: Luc Avérous

Received: 22 February 2021
Accepted: 15 March 2021
Published: 20 March 2021

Publisher's Note: MDPI stays neutral with regard to jurisdictional claims in published maps and institutional affiliations.

Copyright: © 2021 by the authors. Licensee MDPI, Basel, Switzerland. This article is an open access article distributed under the terms and conditions of the Creative Commons Attribution (CC BY) license (http://creativecommons.org/licenses/by/4.0/).

Table 1. The full list of mechanical properties obtained from the tensile, flexural and Charpy impact measurements.

	Tensile Test			Flexural Test		Charpy Test
	**Modulus (MPa)**	**Strength (MPa)**	**Elongation at Break (%)**	**Modulus (MPa)**	**Strength (MPa)**	**Impact Strength (kJ/m²)**
**Blends—Injection Molding**						
PLA	2880 (±124)	62.7 (±0.1)	3.9 (±0.1)	3540 (±250)	83.3 (±1.9)	1.9 (±0.4)
PLA-PBAT10	2330 (±142)	56.9 (±1.9)	11.0 (±2.7)	3120 (±243)	96.8 (±4.9)	2.3 (±0.1)
PLA-PBAT20	2190 (±58)	50.3 (±0.9)	45.0 (±11.0)	2800 (±135)	87.6 (±2.9)	3.1 (±0.1)
PLA-PBAT30	2020 (±39)	41.7 (±1.3)	195.0 (±42.0)	2590 (±114)	79.1 (±2.7)	4.0 (±0.1)
PLA-PBAT10-CE	2750 (±68)	56.1 (±0.7)	41.0 (±8.6)	2930 (±83)	93.3 (±4.2)	2.4 (±0.3)
PLA-PBAT20-CE	2300 (±417)	48.4 (±0.7)	55.0 (±9.0)	2530 (±102)	81.6 (±1.4)	2.7 (±0.1)
PLA-PBAT30-CE	2280 (±37)	43.4 (±0.4)	237.0 (±35.0)	2450 (±77)	74.5 (±2.0)	4.4 (±0.8)
**Composites—Compression Molding**						
PLA	5470 (±323)	49.6 (±2.2)	2.3 (±0.1)	6655 (±91)	105.0 (±7.1)	6.3 (±0.9)
PLA-PBAT10	4460 (±471)	59.6 (±5.5)	3.9 (±0.5)	6640 (±104)	89.2 (±8.3)	7.9 (±1.1)
PLA-PBAT20	5690 (±240)	52.0 (±5.2)	2.8 (±0.2)	6370 (±146)	74.7 (±2.9)	10.4 (±1.2)
PLA-PBAT30	4577 (±39)	61.0 (±3.3)	5.3 (±0.4)	5570 (±236)	65.8 (±4.7)	9.0 (±0.4)
PLA-PBAT10-CE	6910 (±68)	72.1 (±2.4)	2.4 (±0.1)	7320 (±1040)	101.0 (±8.4)	7.2 (±0.5)
PLA-PBAT20-CE	6280 (±417)	58.9 (±3.0)	2.8 (±0.1)	6950 (±350)	77.2 (±3.0)	9.1 (±1.6)
PLA-PBAT30-CE	6560 (±37)	66.6 (±5.4)	2.7 (±0.4)	5620 (±370)	83.0 (±4.0)	11.3 (±2.2)

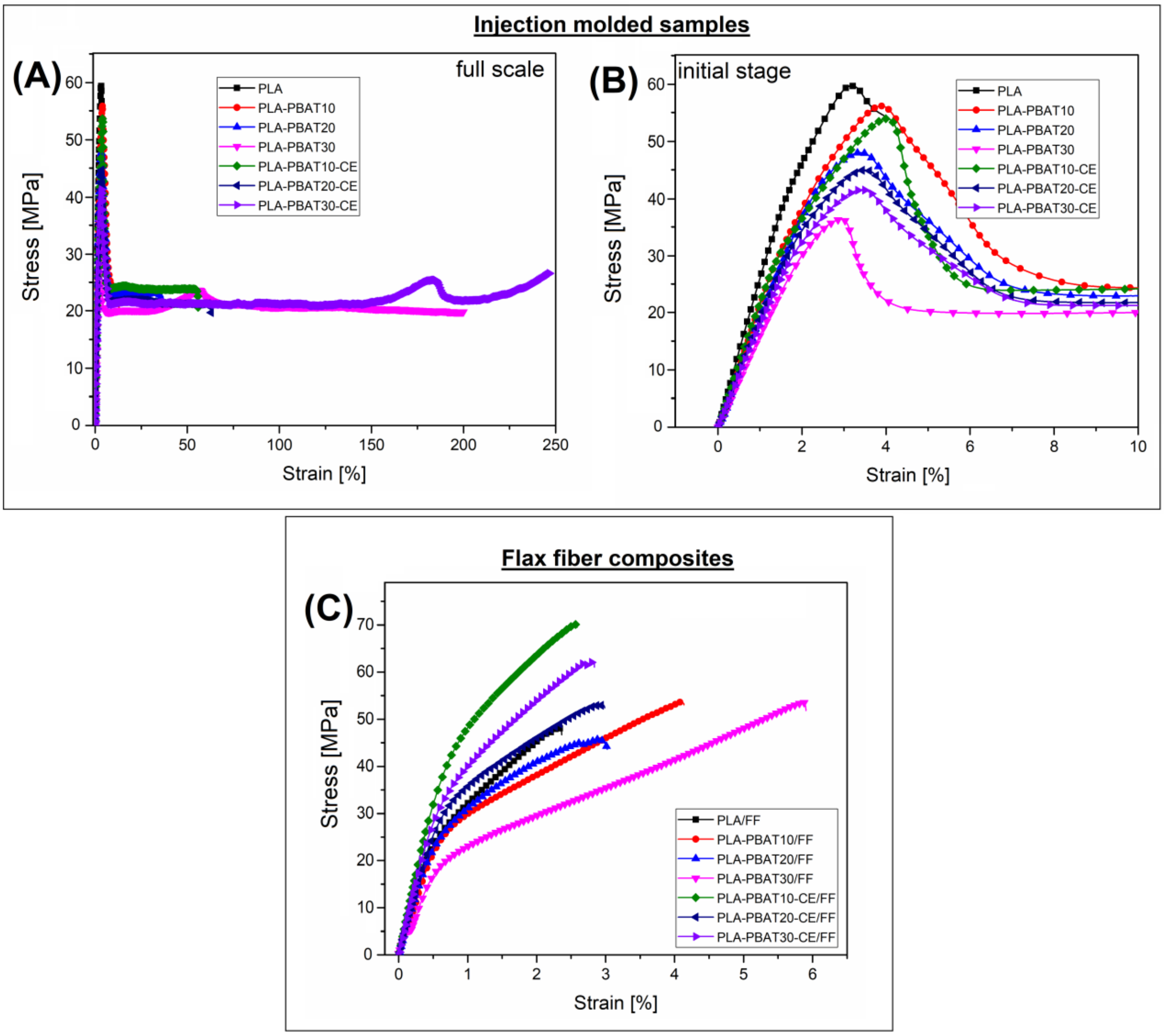

Figure S1. The macroscopic appearance of the 3D printed samples, pictures are presenting the impact test specimens after the fracture.
